# Supplementary material for: Work-Related Psychosocial Factors and Global Cognitive Function: Are Telomere Length and Low-Grade Inflammation Potential Mediators of This Association?
Source: Int J Environ Res Public Health. 2023 Mar 10;20(6):4929. doi: 10.3390/ijerph20064929 (PMC10049148; doi:10.3390/ijerph20064929)
Supplement: Supplementary file 1 [file ijerph-20-04929-s001.zip › ijerph-2199175-supplementary.pdf]

Supplementary Materials

Table of contents

Supplementary methods for statistical analysis

Table S1. Characteristics at T1 of participants randomly selected for blood sample (n=3411) and those not selected (n=5570).

Table S2. Comparison of the characteristics between included and censored participants at T1.

Figure S1. Association between telomere length and cognitive function according to inflammatory index.

Supplementary methods for statistical analysis

Multiple imputation (MI) was done using chained equations (White, Royston, & Wood, 2011) for missing data in T1 variables for 3618 participants (3411 participants randomly selected for the biomarker study plus 207 participants not included in the random selection). The predictor variables included in the MI model were sex, education, smoking status, diabetes, cardiovascular diseases (CVD), hypertension, relationship with children, relationship with spouse, occupation, income, scores of psychological demand, job control and social support at work using the MONICA questionnaire, age, body mass index (BMI), waist-to-hip ratio (WHR), alcohol, physical activity, number of confidants, number of helpers, total cholesterol blood concentrations and systolic blood pressure. Furthermore, interaction terms were included between sex and i) occupation, ii) education, iii) psychological demand score, iv) job control scire, and v) age; between age and i) psychological demand score, and ii) job control score; between occupation and psychological demand score and ii) job control score; and between education and psychological demand score and ii) job control score. Linear models were used for continuous variables and logistic models were used for binary or categorical variables. Sixty MIs were done because 60% of the study population had at least one missing datum on some variables.(White et al., 2011) The second step of MI was done for 3180 participants that were selected for the study on biomarkers and were either present at T2 data collection (n=3087), or not present at T2 but present for the in-person data collection at T3 with a cognitive function measurement (n=93). The same predictor variables were included in this second step with the addition of T2 data for these variables plus other predictor variables that were only assessed at T2, namely: psychological distress, stressful life-events, score of social support at work, reward score, and score of overcommitment at work (Siegrist & Montano, 2013). In this step, one imputation was done for each of the 60 databases created at the first step. The final step of MI was done for the 2219 participants included in the present study. The same procedure as for the second step was used, adding predictor variables that were only assessed at T3: depressive symptoms, self-reported use of anti-inflammatory medication, antecedent of commotion, self-reported arthritis, dietary inflammatory index, Montreal cognitive assessment (MoCA) score, telomere length, and C-reactive protein (CRP) and interleukine-6 (IL-6) concentrations. Interaction terms were also added between telomere and i) sex, ii) age, iii) CRP and iv) IL-6; between CRP and i) sex and ii) age; and between IL-6 and i) sex and ii) age.

Inverse probability of censoring weighting (IPCW) was done to correct for the differences in the characteristics between included participants (n=2219) and those lost to follow-up between each measurement time for each imputed dataset. IPCWs were calculated using predicted values obtained from logistic regressions of the probability of being censored between T1 and T2, and between T2 and T3, according to exposure and specific covariates at T1, and at T1 and T2, respectively (Hernán & Robins, 2020). The covariates included in the model for the probability of being censored between T1 and T2 were age, sex, education smoking status, diabetes, CVD, hypertension, relationship with children, relationship with spouse, occupation, income, scores of psychological demand, job control and social support at work using the MONICA questionnaire,(Lowel et al., 2005) BMI, WHR, alcohol, physical activity, number of confidants, number of helpers, total cholesterol blood concentration and systolic blood pressure, all measured at T1. The same variables were included in the model for the probability of being censored between T2 and T3 but using T2 measurements. Psychological distress, stressful life-events, and scores of social supports at work from the demand-control-support model, of reward, and of overcommitment at work were also added in this model. Final weights were calculated by multiplying together the IPCWs obtained from each of the regression models.

We selected covariates that might confound or modify the evaluated associations. We chose T1 values to respect the temporal sequence of events and minimize overadjustment, with the exception of education, for which T3 values were used. At T3, education was estimated during a face-to-face interview, by a trained interviewer, and evaluated in number of years of education, as compared to T1 and T2 measures which were categorical, thus more likely to introduce residual confounding. These covariates were: age, sex, smoking status, alcohol intake, physical activity, diabetes, cardiovascular diseases, hypertension, BMI, WHR ratio, and social support outside work (number of confidants, number of helpers, relationship with spouse, and relationship with children).

Causal mediated effects of each work-related psychosocial factor exposure were computed using the mediation method proposed by Vansteelandt and Daniel.(Vansteelandt & Daniel, 2017) In this method of analysis, the total effect of exposure is decomposed into a direct component, indirect components through each mediator and a residual indirect component. To estimate these four components, Vansteelandt and Daniel suggest specifying four loss to follow-up weighted models : 1) model of MoCA score as a function of psychosocial stressors at work, controlling for all covariates described above and for the mediators and their interactions (telomere length, inflammatory index, the square of the inflammatory index, and interaction terms between telomere and i) inflammatory index and ii) the square of inflammatory index); 2) model of telomere length as a function of psychosocial stressors at work, controlling for all the covariates; 3) model of inflammatory index as a function of psychosocial stressors at work, controlling for all the covariates; and 4) model of telomere length as a function of psychosocial stressors at work, controlling for the inflammatory index, the square of inflammatory index, and for all other covariates. Direct, indirect and residual effects are then computed from these estimated models using the formulas presented in Vansteelandt and Daniel.(Vansteelandt & Daniel, 2017) Standard errors for these estimated interventional effects are obtained by bootstrap with 199 replications. Weights were computed for each bootstrap sample. This procedure was applied to each imputed dataset and results were combined producing the average interventional effects and Wald-type confidence intervals.

The procedure to estimate direct and indirect effects was implemented in R 4.1.3; all other analyses were performed using SAS software version 9.4.

References

1. Hernán, M. A., & Robins, J. M. (2020). Causal Inference: What If. In Boca Raton: Chapman & Hall/CRC (Ed.). Retrieved from https://cdn1.sph.harvard.edu/wp-content/uploads/sites/1268/2020/02/ci_hernanrobins_21feb20.pdf
2. Siegrist, J., & Montano, D. (2013). ERI-Questionnaires - Users Guide, version 22.11.2012. Germany
3. Vansteelandt, S., & Daniel, R. M. (2017). Interventional Effects for Mediation Analysis with Multiple Mediators. Epidemiology, 28(2), 258-265. doi:10.1097/ede.0000000000000596
4. White, I. R., Royston, P., & Wood, A. M. (2011). Multiple imputation using chained equations: Issues and guidance for practice. Statistics in Medicine, 30(4), 377-399. doi:10.1002/sim.4067

Table S1. Characteristics at T1 of participants randomly selected for blood sample (n=3411) and of those not selected (n=5570).

	**Selected for blood sample N=3411 Mean ± SD or N (%)**	**Not selected N=5570 Mean ± SD or N (%)**
**Age (years)**	39.1 ± 8.7	40.7 ± 8.5
**Sex**		
Males	1639 (48.1)	2850 (51.2)
Females	1772 (52.0)	2720 (48.8)
**Education level completed**		
High school or less	969 (28.7)	1681 (30.4)
College	979 (29.0)	1524 (27.5)
University	1428 (42.3)	2328 (42.1)
Missing	35 (-)	37 (-)
**Occupation**		
Office workers	1123 (33.0)	1804 (32.5)
Technicians	682 (20.1)	1080 (19.4)
Professionals	1181 (34.7)	1873 (33.7)
Managers	306 (9.0)	621 (11.2)
Others	110 (3.2)	177 (3.2)
Missing	9 (-)	15 (-)

**Hours worked per week**		
<35	202 (5.9)	320 (5.8)
35-40	2958 (86.9)	4792 (86.3)
>40	245 (7.2)	444 (8.0)
Missing	6 (-)	14 (-)
**Years worked in the same job**	9.7 ± 8.5	10.9 ± 8.8
Missing	54 (-)	2 (-)
**Smoking status**		
Never smoking regularly	1498 (44.2)	2318 (42.0)
Former regular smoker	1099 (32.5)	1926 (34.9)
Current occasional smoker	161 (4.8)	258 (4.7)
Current regular smoker	628 (18.6)	1018 (18.4)
Missing	25 (-)	50 (-)
**Alcohol intake (drinks/week)**	3.5 ± 4.7	3.6 ± 4.7
Missing	11 (-)	40 (-)
**Physical activity (times/month)**	5.3 ± 4.7	5.2 ± 4.4
Missing	6 (-)	17 (-)
**Body mass index (kg/m^2)**	24.6 ± 4.0	24.7 ± 4.0
Missing	13 (-)	26 (-)
**Waist-to-hip ratio**	0.84 ± 0.1	0.85 ± 0.1
Missing	40 (-)	59 (-)
**Diabetes (yes)**	67 (2.0)	117 (2.1)
Missing	2 (-)	8 (-)
**Hypertension (yes)**	587 (17.2)	1098 (19.7)
**Cardiovascular diseases**	84 (2.5)	165 (3.0)
**Number of confidants**	2.1 ± 1.5	2.0 ± 1.4
Missing	9 (-)	27 (-)
**Number of helpers**	2.9 ± 1.6	2.9 ± 1.6
Missing	9 (-)	14 (-)
**Relationship with spouse**		
Satisfying	2064 (63.5)	3298 (62.0)
Unsatisfying	303 (9.3)	547 (10.3)
No spouse	883 (27.2)	1467 (27.7)
Missing	161 (-)	267 (-)
**Relationship with children**		
Satisfying	1908 (57.9)	3251 (60.6)
Unsatisfying	94 (2.8)	146 (2.7)
No children	1291 (39.2)	1968 (36.7)
Missing	118 (-)	205 (-)
**High psychological demands**	1407 (41.5)	2325 (42.1)
Missing	24 (-)	48 (-)
**Low job control**	2182 (64.4)	3470 (62.8)
Missing	24 (-)	46 (-)
**Job strain**		
Low strain	521 (15.4)	864 (15.7)
Passive	1455 (43.1)	2322 (42.2)
Active	681 (20.2)	1184 (21.5)
High strain	720 (21.3)	1135 (20.6)
Missing	34 (-)	65 (-)

[1]207 participants were not included in the random selection either because they died between T1 and T2 or because they were not traceable for data collection at T3. Mean ± SD or n (%).

Table S2. Comparison of the characteristics[a] between included and censored participants at T1[b.]

	Included<br>N=2219[c]<br>Mean ± SD or N (%)	Censored<br>N=1192[d]<br>Mean ± SD or N (%)
**Age** (years)	38.9 ± 7.9	39.4 ± 10.0
**Sex**		
Males	1086 (48.9)	553 (46.4)
Females	1133 (51.1)	639 (53.6)
**Educational level completed**		
High school or less	553 (25.1)	416 (35.4)
College	652 (29.6)	327 (27.9)
University	997 (45.3)	431 (36.7)
Missing	17 (-)	18 (-)
**Occupation**		
Office workers	646 (29.2)	477 (40.2)
Technicians	477 (21.5)	205 (17.3)
Professionals	816 (36.9)	365 (30.7)
Managers	221 (10.0)	85 (7.2)
Others	54 (2.4)	56 (4.7)
Missing	5 (-)	4 (-)
**Smoking status**		
Never smoking regularly	1012 (46.0)	486 (40.9)
Former regular smoker	735 (33.4)	364 (30.7)
Current occasional smoker	101 (4.6)	60 (5.1)
Current regular smoker	351 (16.0)	227 (23.3)
Missing	20 (-)	5 (-)
**Alcohol intake** (drink/week)	3.5 ± 4.5	3.5 ± 5.0
Missing	2 (-)	9 (-)
**Physical activity** (times/month)	5.3 ± 4.3	5.3 ± 4.5
Missing	4 (-)	2 (-)
**Body mass index** (kg/m^2)	24.5 ± 3.8	24.9 ± 4.4
Missing	9 (-)	4 (-)
**Waist-to-hip ratio**	0.84 ± 0.1	0.84 ± 0.10
Missing	28 (-)	12 (-)
**Diabetes**	34 (1.5)	33 (2.8)
Missing	1 (-)	1 (-)
**Hypertension**	347 (15.6)	240 (20.1)
**Cardiovascular diseases**	52 (2.3)	32 (2.7)
**Number of confidants**	2.1 ± 1.5	2.1 ± 1.4
Missing	4 (-)	5 (-)
**Number of helpers**	3.0 ± 1.6	2.9 ± 1.6
Missing	4 (-)	5 (-)
**Relationship with spouse**		
Satisfying	1370 (64.8)	694 (61.0)
Unsatisfying	215 (10.2)	88 (7.7)
No spouse	528 (25.0)	355 (31.2)
Missing	106 (-)	55 (-)
**Relationship with children**		
Satisfying	1311 (61.1)	597 (52.1)
Unsatisfying	64 (3.0)	30 (2.6)
No children	771 (35.9)	520 (45.3)
Missing	73 (-)	45 (-)
**Passive work at T2**	722 (34.4)	380 (40.6)
Missing	119 (-)	257 (-)
**High job strain at T2**	396 (18.9)	160 (17.1)
Missing	119 (-)	257 (-)
**High psychological demands at T2**	991 (46.9)	399 (42.1)

Missing	104 (-)	425 (-)
**Low job control at T2**	1120 (53.3)	543 (57.8)
Missing	117 (-)	253 (-)
**Low social support at work at T2**	1152 (55.2)	542 (58.0)
Missing	132 (-)	257 (-)
**Effort-reward imbalance**	497 (24.3)	202 (22.6)
Missing	173 (-)	299 (-)
**Low reward**	954 (46.6)	396 (44.2)
Missing	170 (-)	295 (-)
**Iso-strain**	275 (13.3)	120 (13.0)
Missing	146 (-)	271 (-)

[a] If not otherwise mentioned. [b] Participants randomly selected for blood sample. [c] Participants selected for blood sample who had participated at T3, without missing data on cognitive function. [d] Participants censored because of death, lost to follow-up, not traceable or missing data on cognitive function.

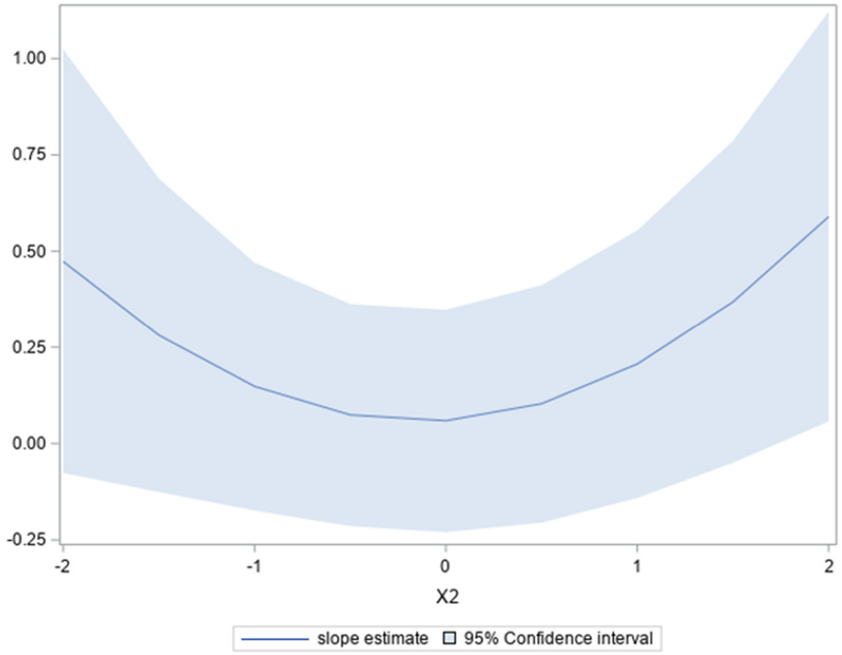

Figure S1. Association between telomere length and cognitive function according to inflammatory index. Y axis = Slope estimates, i.e., adjusted[a] coefficient for the association between telomere length and cognitive performance. X axis =Inflammatory index value (X2). P-value for quadratic interaction = 0.0146. [a]Adjusted for age, sex, education, BMI, smoking, alcohol, physical activity, diabetes, hypertension, cardiovascular diseases, number of confidants, number of helpers, relationship with spouse, relationship with children, inflammatory index, the inflammatory index squared, and including interaction terms between telomere length and inflammatory index and between telomere length and inflammatory index squared.
